# Deep Learning-Based Inverse Design of Stochastic-Topology Metamaterials for Radar Cross Section Reduction

**DOI:** 10.3390/ma18214841

**Published:** 2025-10-23

**Authors:** Chao Zhang, Chunrong Zou, Shaojun Guo, Yanwen Zhao, Tongsheng Shen

**Affiliations:** 1 School of Electronic Science and Engineering, University of Electronic Science and Technology of China, Chengdu 611731, China; chaozhanguestc@std.uestc.edu.cn (C.Z.);; 2 National Institute of Defense Technology Innovation, Academy of Military Sciences PLA China, Beijing 100071, China

**Keywords:** stochastic topology, electromagnetic metamaterials, VAE-CBAM, encoder-only, RCS reduction

## Abstract

Electromagnetic (EM) metamaterials have a wide range of applications due to their unique properties, but their design is often based on specific topological structures, which come with certain limitations. Designing with stochastic topologies can provide more diverse EM properties. However, this requires experienced designers to search and optimise in a vast design space, which is time-consuming and requires substantial computational resources. In this paper, we employ a deep learning network agent model to replace time-consuming full-wave simulations and quickly establish the mapping relationship between the metamaterial structure and its electromagnetic response. The proposed framework integrates a Convolutional Block Attention Module-enhanced Variational Autoencoder (CBAM-VAE) with a Transformer-based predictor. Incorporating CBAM into the VAE architecture significantly enhances the model’s capacity to extract and reconstruct critical structural features of metamaterials. The Transformer predictor utilises an encoder-only configuration that leverages the sequential data characteristics, enabling accurate prediction of electromagnetic responses from latent variables while significantly enhancing computational efficiency. The dataset is randomly generated based on the filling rate of unit cells, requiring only a small fraction of samples compared to the full design space for training. We employ the trained model for the inverse design of metamaterials, enabling the rapid generation of two cells for 1-bit coding metamaterials. Compared to a similarly sized metallic plate, the designed coding metamaterial radar cross-section (RCS) reduces by over 10 dB from 6 to 18 GHz. Simulation and experimental measurement results validate the reliability of this design approach, providing a novel perspective for the design of EM metamaterials.

## 1. Introduction

Electromagnetic (EM) metamaterials can realise precise control over the absorption and scattering of electromagnetic waves by designing effective structural units. They find widespread applications in energy conversion, sensors, and cloaking technology [1,2,3,4]. In recent years, coding metamaterials have attracted much attention because they can realise more precise and flexible control of EM waves by designing specific coding sequences [5,6]. Traditional design methods typically involve unit structure design based on design objectives and coding schemes, parameter scanning using electromagnetic simulation software, and optimisation and verification. However, this design approach is highly complex, requiring significant time and computational resources [7]. Moreover, it heavily relies on the designer’s expertise, exhibits limited topological structure diversity, and imposes restrictions on the design space, making it potentially inadequate for specific design requirements.

To enhance design freedom, stochastic-topology-based metamaterial design methods have been employed [8]. By algorithmically generating rapidly stochastic topological structures, diverse and unique metamaterial configurations can be created, exhibiting distinct EM properties. However, optimising the structure to meet the design objectives becomes more time-consuming as design freedom increases. Many researchers have utilised evolutionary algorithms, such as particle swarm optimisation (PSO) and genetic algorithms (GA), to search for global optimal solutions within the design space through heuristic updating rules [9,10]. These algorithms are easy to implement but still require iterations with simulation software, and the computational cost grows exponentially as the dimensionality of the design variables increases.

Currently, due to the superior performance of deep learning, several scholars have integrated deep learning with metamaterials design, proposing various deep learning-based inverse design methods [11,12,13]. This category of research primarily encompasses two aspects: EM structure generation and EM response prediction. In EM structure generation models, generative adversarial networks (GAN) produce metamaterial unit structures with high visual fidelity through adversarial training, where the generator synthesises complex topologies and the discriminator evaluates structural rationality [14,15,16]. However, this necessitates a substantial amount of training data, particularly for structures with high design freedom such as stochastic-topology metamaterials. The variational autoencoder (VAE) comprises an encoder and a decoder, featuring a simple architecture that facilitates efficient training [17,18]. This model has gained widespread adoption in the design of EM structures in recent years [19,20]. The encoder compresses structural information into a low-dimensional latent space, with sampled vectors subsequently reconstructed or generated by the decoder. Among numerous related studies, further research is required to explore how to improve reconstruction quality and enhance the model’s feature extraction capability. For EM response prediction, latent space variables serve as inputs, while spectral sequences constitute the outputs [21]. The Transformer model, as a core architecture for sequence modelling, has extensive applications in natural language processing, time-series forecasting, and cross-modal learning [22]. The original Transformer employs an encoder-decoder architecture that treats spectral sequences as temporally dependent outputs, which easily leads to error accumulation. In contrast, the encoder-only configuration establishes direct mappings between latent variables and spectral sequences through global self-attention mechanisms, thereby eliminating redundant decoder modules to significantly boost computational efficiency [23].

In summary, this work proposes a neural network-based assisted inverse design algorithm for rapid design of stochastic topological metamaterials [24,25]. The novelty of our approach is its integrated deep learning framework, which provides an efficient and scalable solution for the inverse design of stochastic-topology metamaterials by synergizing a CBAM-enhanced VAE with an encoder-only Transformer. Specifically, this methodology integrates a convolutional block attention module (CBAM) [26] into the VAE framework to enhance feature extraction capability, while employing an encoder-only architecture for predicting electromagnetic response amplitude and phase of stochastically configured materials. This approach maintains modelling capacity while significantly improving computational efficiency. Furthermore, we implement a multi-task weighted loss function to balance structural reconstruction and response prediction. Ultimately, the proposed network is used to replace simulation software for the design of 1-bit coding metamaterials, achieving a Radar Cross Section (RCS) reduction of more than 10 dB across the 6–18 GHz frequency range.

## 2. Theories and Methods

### 2.1. Stochastic Topology Metamaterials

**Figure 1:**
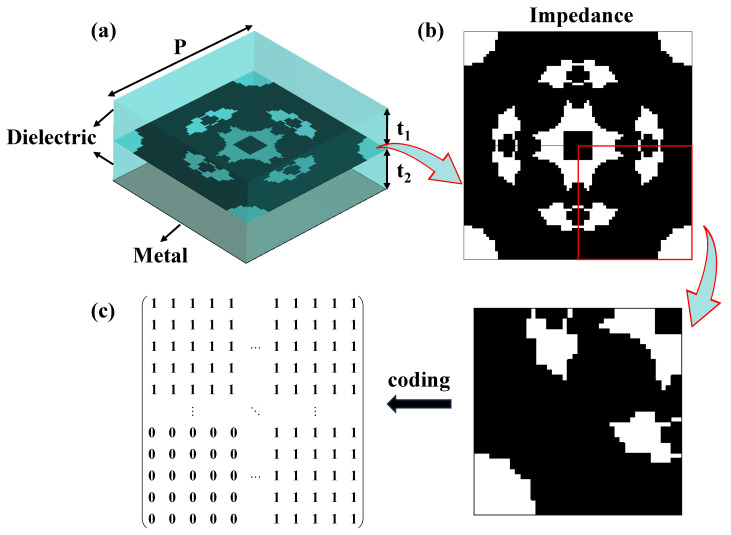
Schematic of the metamaterial unit cell and its coding rules: (**a**) the layered structure of the unit cell, from top to bottom: a dielectric layer, an impedance layer, a second dielectric layer, and a metallic plate; (**b**) the topology of the impedance layer; (**c**) the structural coding rules.

In this work, as shown in Figure 1a, the stochastic topology metamaterial unit consists of four layers: dielectric-impedance-dielectric-metal, with a unit cell period *P* = 8 mm. The upper and lower dielectric layers are made of F4BTM300 with a relative permittivity of *ε*_r_ = 3.0 and a loss tangent of tan *θ* = 0.001, both having a thickness of *t*_1_ = *t*_2_ = 3.5 mm. The impedance layer, implemented using a nickel-chromium (NiCr) thin-film (Ticer TCR^®^, Ticer Technologies) with a sheet resistance of 100 ohm/sq, follows the p4m rule [8], resulting in an axisymmetric structure as illustrated in Figure 1b. Subsequently, the impedance layer structure is encoded using “1” and “0” to represent the presence or absence of the impedance film, following the coding rules depicted in Figure 1c. The entire structure is divided into *N* × *N* small cells. Since the generated image exhibits an axisymmetric structure, a randomly generated coding matrix with a size of *N*/2 × *N*/2 is flipped and superimposed, followed by two symmetries, to generate the complete structure. The bottom conductor is composed of a copper foil with a thickness of *t*_*pec*_ = 0.017 mm.

### 2.2. Deep Learning Approach

**Figure 2:**
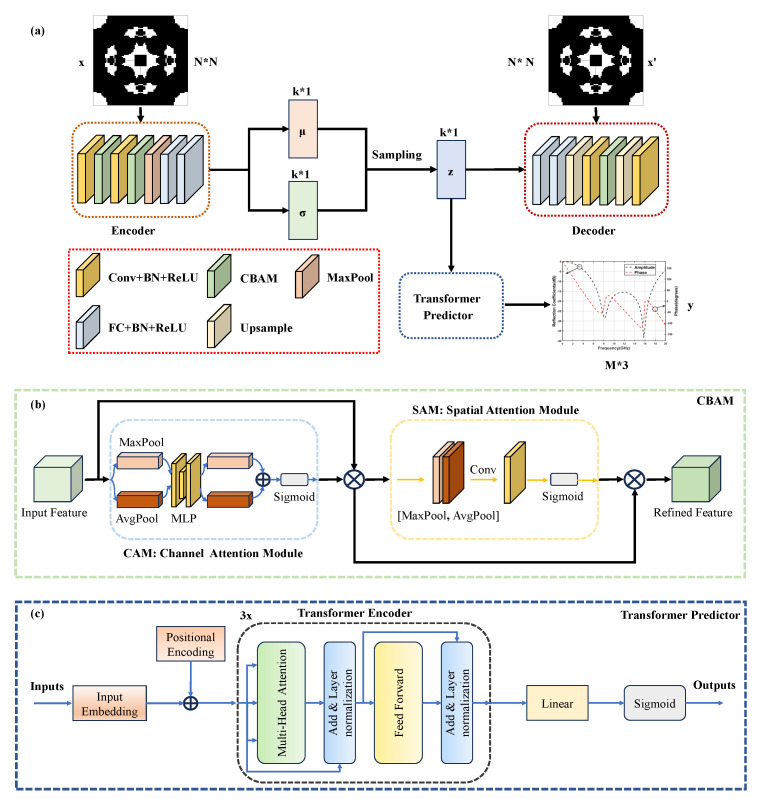
Schematic of the multi-task deep learning network. (**a**) Overall architecture of CBAM-VAE-Transformer network; (**b**) Structure of CBAM attention mechanism; (**c**) Configuration of Transformer-based predictor.

To achieve topology reconstruction and EM response prediction, we architected a multi-task deep learning network. As illustrated in Figure 2, the framework integrates a CBAM-enhanced VAE module and a Transformer predictor, respectively performing metamaterial structural reconstruction and electromagnetic response prediction.

#### 2.2.1. CBAM-VAE

The VAE is a deep learning model that integrates Bayesian networks, primarily comprising an encoder and a decoder, as depicted in Figure 2a [27]. Here, *x* denotes the input stochastic topological structure, *z* represents the latent variable, and *x*′ signifies the reconstructed or generated stochastic topological structure. The encoder first maps the high-dimensional input *x* to an approximate posterior distribution $q_{\phi }\left(z|x\right)$ over the latent variables *z*, assumed to follow a normal distribution,

(1) $$q_{\phi }\left(z|x\right)=N\left(z;\mu ,\sigma^{2}I\right)$$

where μ and $\sigma^{2}$ denote the mean and variance of the normal distribution, *I* is the identity matrix, and ϕ represents variational parameters. Since the relationship between latent variable *z* and parameters ϕ is non-deterministic, directly sampling *z* from the encoder is non-differentiable, preventing gradient backpropagation. To resolve this, a stochastic sample ε is introduced, enabling differentiable sampling of latent variable *z* via the reparameterization trick,

(2) z=μ+σ⊙ε

where ε follows a standard normal distribution N(0,I). Finally, the decoder reconstructs the latent variable *z* into *x*′.

To enhance the feature extraction and reconstruction capabilities of the VAE model, CBAM is introduced. As a lightweight attention module that combines channel and spatial dimensions, CBAM comprises two submodules: the channel attention module (CAM) and the spatial attention module (SAM), as shown in Figure 2b. For an intermediate feature map, CBAM sequentially infers attention maps along two separate dimensions (channel and spatial), then multiplies these maps with the input feature map for adaptive feature refinement.

The CAM aggregates spatial information through concurrent global average pooling (AvgPool) and max pooling (MaxPool), processed via a shared multilayer perceptron (MLP) to generate a 1D channel attention vector. This operation compresses spatial dimensions without altering channel depth, focusing on semantically meaningful information within the input feature map. The channel attention mechanism can be expressed as:

(3) $$\begin{array}{cc} M_{c}\left(F\right) & =\delta (MLP(AvgPool(F))+MLP(MaxPool(F)))\\ & =\delta (W_{1}^{m}\left(W_{0}^{m}\left(F_{\mathrm{Avg}\;}^{c}\right)\right)+W_{1}^{m}\left(W_{0}^{m}\left(F_{\mathrm{Max}\;}^{c}\right)\right)) \end{array}$$

where $M_{c}\left(F\right)$ denotes the CAM output, and δ represents the sigmoid operation. $W_{1}^{m}$ and $W_{0}^{m}$ indicate weights of the two-layer MLP.

Subsequently, the SAM processes the channel-refined feature map through separate average pooling (AvgPool) and maximum pooling (MaxPool) operations along the channel axis. The resulting outputs are concatenated and processed by a convolution layer to generate a 2D spatial attention map. These element-wise multiplied attention maps are applied to the input feature map for adaptive feature refinement. This module preserves spatial dimensionality while compressing channel depth, focusing on target positional information. The spatial attention mechanism is formulated as:

(4) $$\begin{array}{cc} M_{s}\left(F\right) & =\delta (f^{7\times 7}\left[AvgPool\left(F\right);MaxPool\left(F\right)\right])\\ & =\delta (f^{7\times 7}\left(\left[F_{\mathrm{Avg}\;}^{s};F_{\mathrm{Max}\;}^{s}\right]\right)) \end{array}$$

where $M_{s}\left(F\right)$ denotes the SAM output, and $f^{7\times 7}$ indicates the convolution kernel size. As CBAM is a lightweight general-purpose module, it incurs negligible overhead and can be directly integrated into the VAE architecture.

The CBAM-VAE model training is performed by maximising the evidence lower bound (ELBO), equivalent to minimising the following loss function,

(5) $$\begin{array}{c} L_{VAE}=L_{recon}+L_{KL} \end{array}$$

where $L_{recon}$ denotes the reconstruction error, quantifying the accuracy of decoder output *x*′; and $L_{KL}$ represents the Kullback-Leibler divergence loss, constraining the approximate posterior distribution $q_{\phi }\left(z|x\right)∼N\left(\mu ,\sigma^{2}I\right)$ to converge toward the isotropic Gaussian prior p(z)∼N(0,I). The synergistic optimisation of reconstruction and KL terms enables the model to learn a well-structured latent space while maintaining effective data reconstruction capability.

#### 2.2.2. Transformer Predictor

In the proposed architecture, the transformer predictor module employs an encoder-only configuration to map latent variables *z* to multi-frequency EM response predictions. As illustrated in Figure 2c, the structure comprises an input layer, a transformer encoder, and an output layer. To satisfy the transformer encoder’s input-output sequence length equivalence, the 16-dimensional latent variable *z* is first projected into a 64-dimensional feature space via linear projection, followed by sequence replication to construct an 81-length frequency-position sequence. Learnable positional encodings are incorporated into the input layer and added to the expanded sequence, serving as input to the transformer encoder.

The multi-layer transformer encoder forms the core component, extracting features from latent variable sequences while establishing global dependencies among frequency points. Each encoder layer consists of two sub-layers: a multi-head self-attention (MHSA) layer and a feed-forward network (FFN) layer. Both sub-layers employ residual connections and layer normalisation to stabilise training and prevent gradients from vanishing or exploding. The MHSA mechanism comprises multiple parallel attention heads where input sequences undergo linear transformations to generate query (Q), key (K), and value (V) vectors. Attention outputs from all heads are concatenated and fused through linear projection, computed as follows,

(6) $$\begin{array}{cc} Attention(Q,K,V) & =softmax(\frac{QK^{T}}{\sqrt{d_{k}}})V\\ MultiHead(Q,K,V) & =Concat(head_{1},\cdots ,head_{h})\\ where\;\;\;head_{i} & =Attention(Q_{i},K_{i},V_{i}) \end{array}$$

where $\sqrt{d_{k}}$ denotes the dimensionality of K, Softmax serves as the activation function to normalize attention scores into a probability distribution [28], and Concat is the concatenation operation that combines outputs from multiple attention heads [29]. The FFN employs dual linear transformations with ReLU activation to enhance model representational capacity, formulated as,

(7) $$\begin{array}{c} FFN\left(x\right)=\max\left(0,W_{1}^{l}+b_{1}\right)W_{2}^{l}+b_{2} \end{array}$$

where $W_{1}^{l}$ and $W_{2}^{l}$ are weight matrices, $b_{1}$ and $b_{2}$ denote bias vectors, and max(,) represents the ReLU activation function. Finally, the output layer transforms features at each frequency point into the electromagnetic response domain via linear transformation, while applying a sigmoid activation to constrain outputs to [0,1],

(8) $$\begin{array}{c} S\left(x\right)=\frac{1}{1+e^{-x}} \end{array}$$

### 2.3. Data Gathering and Model Training

In this paper, we employed PyCharm-CST (CST STUDIO SUITE 2023) [30] to conduct joint simulations on metamaterials and obtain EM response curves, thereby enhancing the integration of subsequent deep learning networks. Initially, stochastic-topology structures were generated according to the p4m rule and recorded in a coding form [8]. Subsequently, the generated GDS files were imported into CST for modelling. The simulation utilised a time-domain solver to solve within the 0 to 20 GHz frequency range. The process was fully automated, generating a dataset of 70,000 samples. The full-wave simulation for each unit cell required approximately 1 min, amounting to a total computational time of around 70,000 min. It is specifically noted that our dataset was randomly generated according to unit cell fill ratios to characterise the stochastic topology design space comprehensively [20].

**Figure 3:**
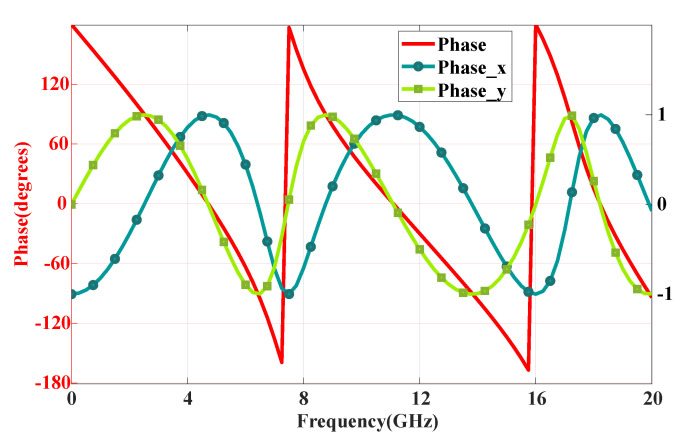
Projecting discontinuous phases on the x− and y− axes of the unit circle.

Next, the generated data underwent preprocessing. To comprehensively describe the information of the stochastic-topology structures, the randomly generated 40 × 40 encoding matrix was expanded to an 80 × 80 matrix, serving as the input for the network structure. Additionally, due to the excessive length of the data exported from CST, we performed sampling to reduce its dimensionality. The sampling interval was set to 25, resulting in a data length of 81 after sampling. Moreover, we addressed the discontinuity of the phase curves by mapping them to the x and y axes of the unit circle, as demonstrated in Figure 3. Finally, the amplitude and phase of the response curves were normalised and utilised as outputs for the deep learning network.

The designed multi-task deep learning network, illustrated in Figure 2a, comprises three distinct objectives: structure reconstruction, KL regularisation, and electromagnetic response prediction. Given their varying learning complexities and convergence rates, a weighted loss function L is employed,

(9) $$\begin{array}{c} \begin{array}{cc} L & =\lambda_{1}L_{recon}+\lambda_{2}L_{KL}+\lambda_{3}L_{pre}\\ L_{recon} & =-\frac{1}{N}\sum_{i=1}^{N}\left(x_{i}log\left(x_{i}^{\prime }\right)+\left(1-x_{i}\right)log\left(1-x_{i}^{\prime }\right)\right)\\ L_{KL} & =\frac{1}{2N}\sum_{i=1}^{N}\left(\sigma_{i}^{2}+\mu_{i}^{2}-log\left(\sigma_{i}^{2}\right)-1\right)\\ L_{pre} & =\frac{1}{N}\sum_{i=1}^{N}\left(y_{i}-y_{i}^{\prime }\right) \end{array} \end{array}$$

where $L_{recon}$ denotes the metamaterial structural reconstruction loss, $L_{KL}$ represents the Kullback-Leibler divergence loss, and $L_{pre}$ signifies the EM response prediction loss. Here, N indicates the sample size, while $\lambda_{1,2,3}$ are hyperparameters that coordinate multi-task optimisation and balance learning priorities.

Phase-dependent weighting dynamically adjusts learning focus: higher initial weights on $L_{recon}$ and $L_{pre}$ capture foundational features, while progressively increasing $L_{KL}$ regularisation in later stages regularises the latent space. This strategy prevents gradient dominance by any single loss term that would degrade other tasks, ultimately achieving a balance between reconstruction fidelity, prediction accuracy, and latent space organisation.

The dataset was randomly split once into a training set (80%) for model development and a held-out test set (20%) for final evaluation. The network was configured with the following parameters: batch_size = 128 and epoch = 120. All computational work, including dataset construction and network training, was performed on a ThinkStation workstation (Windows 11) featuring an NVIDIA Quadro P6000, two Intel Xeon Gold 6154 @ 3.0 GHz CPUs, and 512 GB RAM.

## 3. Result and Discussion

### 3.1. Deep Learning Network

Upon completing training, the developed multi-task deep learning network performs two functions: (1) reconstruction or generation of EM structures, and (2) EM response prediction. To evaluate the trained model’s performance, reconstruction accuracy and mean squared error (MSE) are adopted as quantitative metrics,

(10) $$\begin{array}{cc} recon\_acc & =\frac{1}{N}\sum_{i=1}^{N}I\left(x_{i}=x_{i}^{\prime }\right)\\ pre\_mse & =\frac{1}{M}\sum_{j=1}^{M}{\left(y_{j}-y_{j}^{\prime }\right)}^{2} \end{array}$$

where *N* denotes the total pixel count across all samples, $x_{i}$ and $x_{i}^{\prime }$ represent the *i*-th pixel of the binarised ground-truth and reconstructed images, respectively, and I is the indicator function equaling 1 when the condition holds and zero otherwise. *M* signifies the aggregate frequency points across samples, with $y_{i}$ and $y_{i}^{\prime }$ indicating the *j*-th normalised frequency point value of true and predicted EM responses.

**Figure 4:**
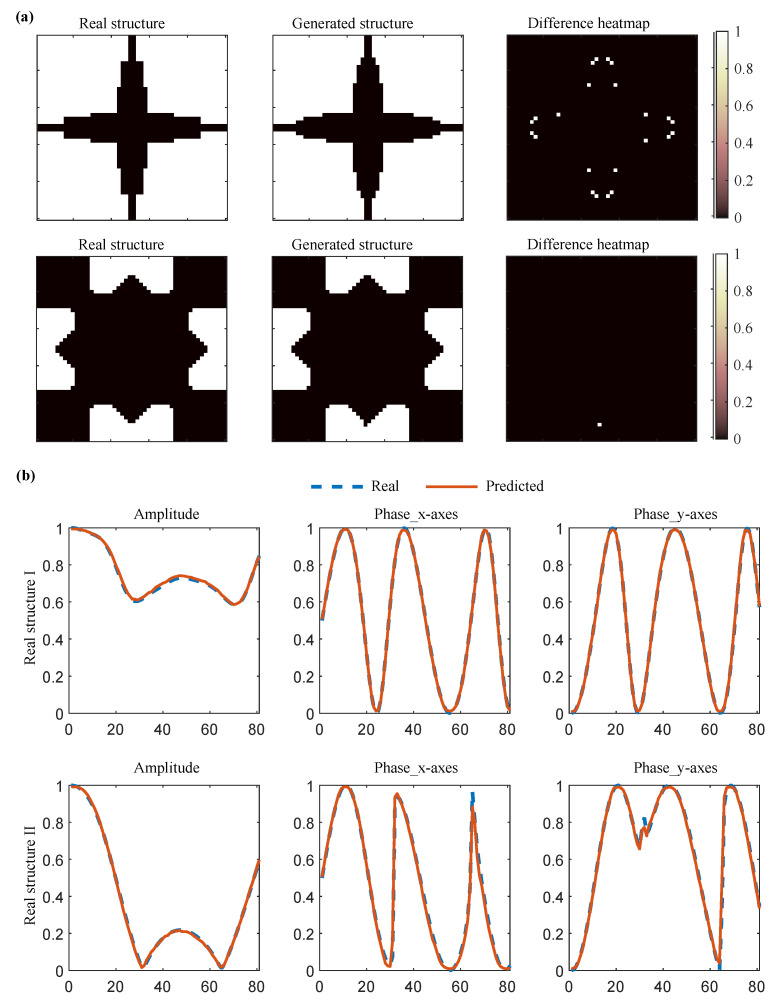
Examples of (**a**) Structural reconstruction and (**b**) EM response prediction.

Testing demonstrates 98% reconstruction accuracy and a prediction error of 1 × 10^−4^ on the test set. Two randomly selected samples, visualised in Figure 4, confirm the precise structural generation alongside accurate amplitude and phase response prediction.

### 3.2. Inverse Design of 1-Bit Coding Metamaterials

**Figure 5:**
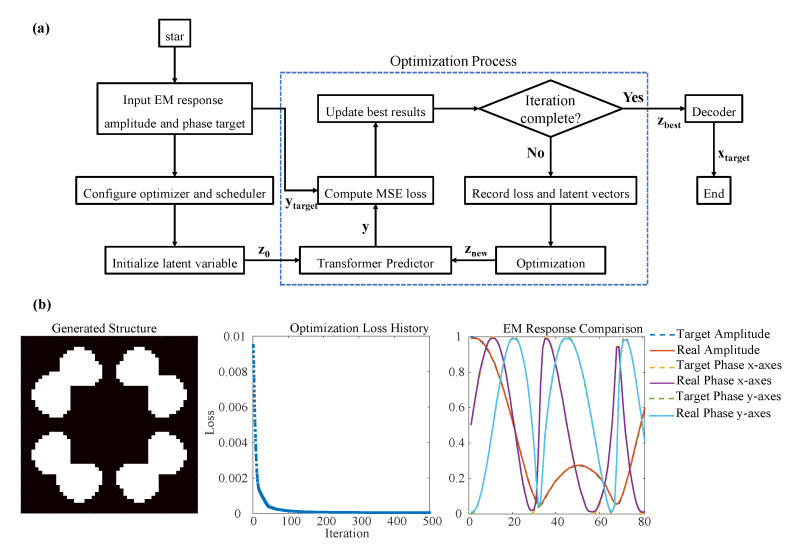
Flowchart and design example of metamaterial inverse design based on a deep learning network. (**a**) Inverse design flowchart; (**b**) Generated structure, optimisation loss, and EM response comparison.

The trained deep learning network enables the efficient generation of stochastic-topology metamaterials that meet specified EM response requirements. As detailed in Figure 5a, the workflow consists of three stages: initialisation, optimisation, and structure generation. During initialisation, the target EM response amplitude and phase $y_{target}$ is defined while the latent variable $z_{0}$ is randomly sampled in latent space. Gradient descent is performed using the Adam optimiser with learning rate scheduling [31].

In the iterative optimisation phase:

(1) The transformer predictor predicts EM responses for current latent variables *y*;
(2) The MSE between predictions and targets is computed;
(3) Optimal latent vector $z_{best}$ is recorded when new minimum MSE occurs;
(4) Latent vectors $z_{new}$ are updated via backpropagation.

Iteration terminates when either the maximum number of iterations (500) is reached and the MSE falls below an acceptable threshold. The optimal $z_{best}$ then generates the final binarised structure $x_{target}$, with its real EM response subsequently calculated.

Figure 5b demonstrates inverse design results for a stochastic specimen. The loss function decreased rapidly during optimization: 1 × 10^−3^ at iteration 25 and 1 × 10^−4^ at iteration 132, converging to 3 × 10^−5^. The entire inverse design process was completed in only 3.2 s on the ThinkStation workstation. This total time includes loading the pre-trained model and executing 500 optimization iterations, representing a speed improvement of several orders of magnitude compared to traditional EM simulation-based methods that would require hundreds of minutes for a comparable design task. A comprehensive assessment must consider the total time investment, including the initial overhead of our deep learning approach. The initial investment requires approximately 1167 h for dataset generation and 1 h for model training. Although substantial, this initial cost is amortized over subsequent designs. A conventional design process relying on 500 sequential full-wave simulations would require approximately 500 min. Analysis indicates that a break-even point is reached when designing more than approximately 140 units. Beyond this point, our method achieves a lower total time investment, with the efficiency advantage becoming substantial for large-scale design tasks.

**Figure 6:**
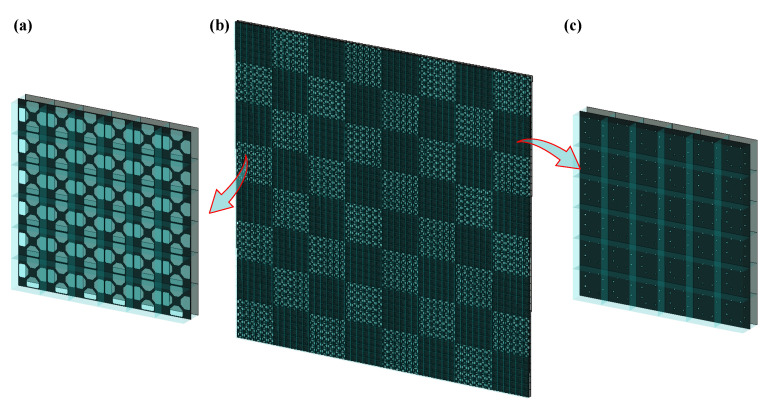
Full model of chessboard metamaterial and its supercells. (**a**) element “0”; (**b**) chessboard metamaterial; (**c**) element “1”.

As an application, we designed a 1-bit coding metamaterial based on stochastic topological structures, where two metamaterial units exhibited a phase difference of 180°. The designed metamaterial units are arranged in a 6 × 6 periodic pattern to form two supercells, labelled as “0” and “1”, as shown in Figure 6. The overall metamaterial consists of an 8 × 8 array of supercells arranged in a chessboard pattern, with dimensions of 384 mm × 384 mm.

**Figure 7:**
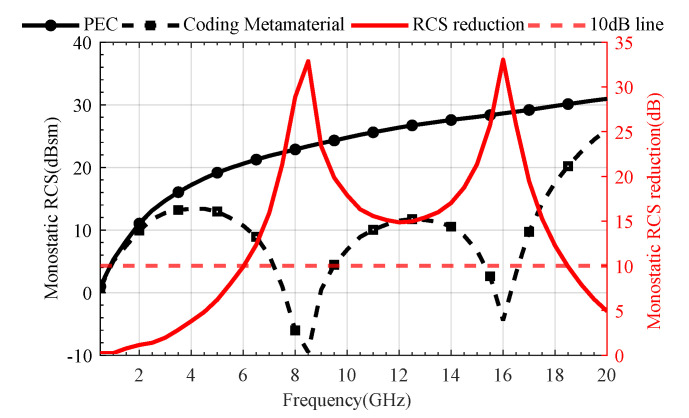
Comparison of RCS reduction simulation results between 1−bit coding metamaterials and metallic plate.

**Figure 8:**
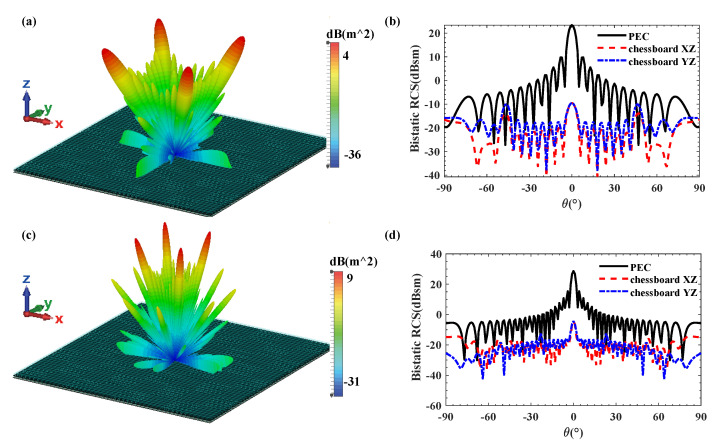
3D bistatic scattered fields at (**a**) 8.5 and (**b**) 16 GHz under normal incidence for the 1-bit coding metamaterial with chessboard structure; Comparison of the bistatic scattered fields along the XZ/YZ planes at (**c**) 8.5 and (**d**) 16 GHz.

Figure 7 illustrates the simulation results of the monostatic RCS and RCS reduction for the designed coding metamaterial compared to a metallic plate of the same size. The coding metasurface achieved a significant RCS reduction of more than 10 dB within the frequency range of 6–18 GHz (100% relative bandwidth). At 8.5 GHz and 16 GHz, the maximum RCS reduction values are obtained, reaching 32.87 dB and 33.05 dB, respectively. Furthermore, Figure 8 presents the 2D and 3D bistatic RCS patterns at 8.5 GHz and 16 GHz. From Figure 8a,c, we can observe that the incident plane wave is scattered into four main beams: *φ* = 45°, 135°, 225° and 315°. This scattering behaviour effectively suppresses RCS. The 2D scattering plots of coding metamaterials at 8.5 and 16 GHz are given in Figure 8b,d. The scattered field is effectively suppressed in all directions compared to the PEC. This is attributed to the coding metamaterial design, which disperses the incident electromagnetic wave energy in all directions.

**Figure 9:**
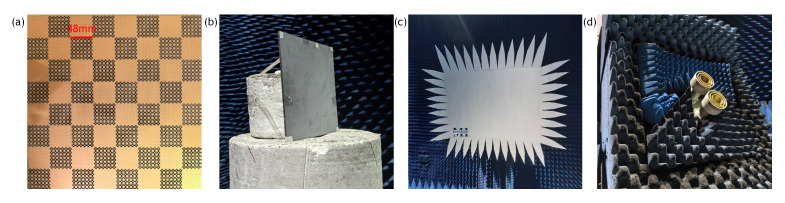
Fabricated chessboard Metamaterial and compact antenna test range (CATR) test environment. (**a**) Fabricated metasurface prototype; (**b**) Target mounted on radar-transparent foam supports; (**c**) Reflector; (**d**) Transmit/receive horn antennas.

To validate the proposed methodology against full-wave simulation results, we fabricated and experimentally characterised the chessboard metamaterial prototype illustrated in Figure 9. The manufactured specimen (384 mm × 384 mm × 7 mm) employs dual-layer F4BM300 substrates ($\varepsilon_{r}=3.0$) with 0.017 mm copper cladding. Figure 9 details the measurement configuration in a compact antenna test range (CATR) chamber, where the prototype was mounted on radar-transparent foam supports. The low-scattering metamaterial was designed for operation in the 6-18 GHz frequency range. However, due to equipment limitations, there was no standard gain horn antenna that directly covers this entire band. Therefore, horn antennas for different frequency segments were used in sections for testing. The frequency bands of the test horn antennas are 5.8–8.2 GHz, 8.2–12.4 GHz, and 12.4–18 GHz, respectively. Each antenna replacement necessitated recalibration and background subtraction. The final full-band response was obtained by stitching the data from these antennas, specifically by averaging the measured values in the overlapping frequency regions (i.e., around 8.2 GHz and 12.4 GHz). Owing to the symmetric configuration of the metamaterial, the structure is polarization-insensitive. Monostatic RCS measurements were performed under co-polarized (HH) excitation at normal incidence.

**Figure 10:**
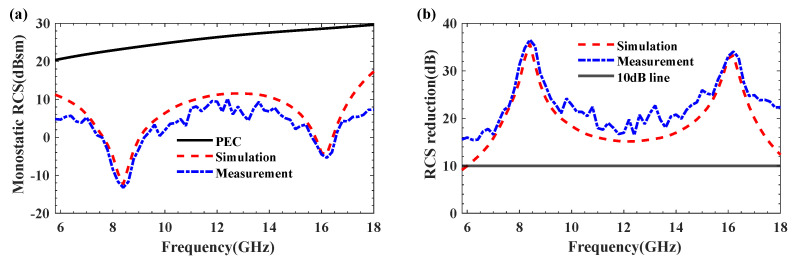
Comparison of simulation and measurement results for monostatic RCS (**a**) and RCS reduction (**b**).

Under HH-polarised wave normal incidence, the comparison between the measured and simulated monostatic RCS results of the low-scattering checkerboard metamaterial as a function of frequency is shown in Figure 10. The designed metamaterial achieves over 10 dB RCS reduction across the entire 6–18 GHz band compared to reference metal plates of equivalent thickness. The acceptable errors may arise from metamaterial fabrication errors or angular deviations between the metamaterial and the reflecting surface.

### 3.3. Discussion

To further validate the superiority and practicality of the proposed methodology, comparative analyses were conducted against prior works in Table 1 and Table 2 focusing on network architecture and RCS reduction performance. Our network enhances the baseline VAE by integrating CBAM, significantly improving feature extraction capability and structural reconstruction fidelity—achieving higher reconstruction accuracy than conventional autoencoder (AE). The encoder-only Transformer predictor attains lower mean squared error (MSE) for electromagnetic response prediction compared to CNN-based models. Crucially, the proposed method exhibits the highest total permutations, yet it requires only a modest increase in training samples while simultaneously predicting the amplitude and phase of EM response. As observed from Table 2, this work employs stochastic topology, offering higher degrees of freedom, and the designed coding metamaterial achieves a maximum fractional bandwidth of 100% in RCS reduction. This finding validates the effectiveness of the proposed method.

## 4. Conclusions

This paper proposes a deep learning network-based rapid design methodology for stochastic-topology metamaterials. The VAE model maps high-dimensional structural information to low-dimensional latent variables, enabling the reconstruction and generation of metamaterials. Incorporating CBAM modules enhances the feature extraction capability, achieving a 98% reconstruction accuracy. An encoder-only Transformer predictor accurately forecasts electromagnetic responses (amplitude and phase) from latent variables with a prediction error of 1 × 10^−4^.

The trained network facilitates inverse metamaterial design, converging to a response error of 3 × 10^−5^ within 500 iterations while consuming only 3.2 s. For demonstration, 1-bit coding metasurfaces were designed, rapidly yielding two unit cells with a 180° phase difference, which achieve an RCS reduction of over −10 dB in the 6–18 GHz range. Simulation and experimental measurements jointly validate the effectiveness of this approach.

Collectively, our framework replaces computationally intensive full-wave simulations in conventional workflows, enabling efficient inverse design and establishing a promising framework for performance-driven metamaterial synthesis. Despite this success, the model’s generalizability beyond stochastic topologies presents a limitation and an opportunity for future work. Looking ahead, we will focus on multi-objective optimization and active learning to further reduce data dependencies, extending this efficient design approach to a broader range of electromagnetic and multi-physics applications.

## Figures and Tables

**Table 1:** Metamaterials Design Methods.

Total Permutations	Algorithm	Training Samples	Target Spectrums	Recon_Loss	Pre_Mse	Reference
2^64^	CNN	24,000	Absorption	-	7.2 × 10^−4^	[19]
2^108^	AE+DLN	18,000	Amplitudes	95%	-	[21]
2^1600^	CBAM-VAE + Transformer	70,000	Amplitudes and phases	98%	1 × 10^−4^	This work

**Table 2:** Metamaterials for RCS Reduction.

Topology	Method	−10 dB RCSR BW (GHz)	FBW	Reference
Single	NA	5.4–14.2	90%	[2]
Single	PSO	7.5–17.5	80%	[32]
Stochastic	DL	6–18	100%	This work

## Data Availability

The raw data supporting the conclusions of this article will be made available by the authors on request.
